# P-1512. EXPLORING KNOWLEDGE AND BARRIERS TO HPV vaccination among mothers of 9-14 years old - A descriptive study in a tertiary care hospital

**DOI:** 10.1093/ofid/ofaf695.1696

**Published:** 2026-01-11

**Authors:** Tara B Nair, C Shiju Kumar, B S Vishnu

**Affiliations:** KIMS TRIVANDRUM, Trivandrum, Kerala, India; Kimshealth hospital, Trivandrum, Kerala, India; Kimshealth hospital, Trivandrum, Kerala, India

## Abstract

**Background:**

Human papilloma virus is pivotal in preventing cervical cancer and other HPV related diseases.However uptake remains suboptimal, partly due to parental knowledge gaps and barriers.

**Figure:**
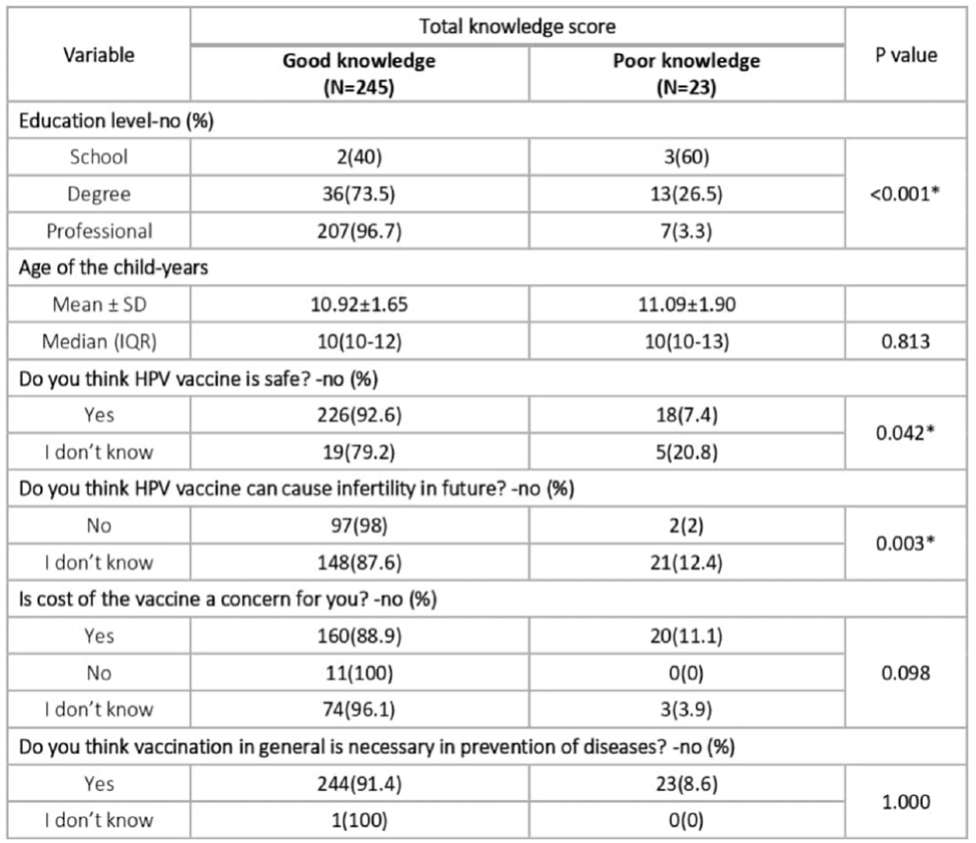

Knowledge score in study population

**Methods:**

This prospective descriptive study was conducted in a tertiary care hospital in Trivandrum, Kerala. A structured preformed questionnaire was administered to total of 268 participants who were mothers of girl children aged 9-14 years attending outpatient Pediatric department. The questionnaire assessed knowledge about HPV,the vaccine available for HPV and factors influencing vaccination.

**Results:**

Among the 268 study subjects, a significant majority,comprising 91.4% demonstrated a commendable level of understanding regarding the HPV vaccine. Various factors were identified as barriers to vaccination uptake, with safety concerns being the most prevalent, cited by 91% of respondents. Additionally a notable percentage (36.9%) expressed uncertainty regarding potential future infertility concerns linked to the vaccine, while 67.2% cited the high costs associated with it as a deterrent.Nevertheless an overwhelming majority (99.6%)acknowledged the indispensable role of vaccines in disease prevention.

**Conclusion:**

The substantial knowledge observed within our study cohort can be credited to the statesman commendable literacy rates and the concerted efforts of governmental and non-governmental entities to fortify healthcare awareness.This study provides valuable insights about the gap in knowledge and measures to augment HPV vaccination rates among adolescents.

**Disclosures:**

All Authors: No reported disclosures

